# What is the quality-of-life status of patients with keratoconus who have not had a surgical intervention? A systematic review

**DOI:** 10.1038/s41433-025-04053-0

**Published:** 2025-10-22

**Authors:** Daliya Sari, Himal Kandel, Richard Kha, Stephanie L. Watson

**Affiliations:** Sydney Medical School, Faculty of Medicine and Health, The University of Sydney, Sydney, NSW Australia

**Keywords:** Corneal diseases, Diseases

## Abstract

The aim was to assess the overall quality of life (QoL) of keratoconic patients who had not undergone surgical interventions by evaluating domain scores such as symptoms, activity limitation, psychological changes, mental health and social wellbeing. The search strategy was conducted using five different search platforms (PubMed, Medline, Scopus, Emcare, CINAHL). We included prospective or retrospective studies assessing the quality of life of patients with keratoconus who have not had a surgical intervention to manage their keratoconus. Data collected were assessed according to their psychometric properties, validity, reliability and responsiveness. Articles were screened for suitability and data were extracted. Bias was assessed with Cochrane RoB2.0 tool and ROBIN – I tool. Six hundred and thirty one results were yielded from the searches and a total of 70 articles were screened based on the inclusion and exclusion criteria. A final total of 26 articles were included. Studies had sample sizes ranging from 25 to 1209 participants with keratoconus. The NEI VFQ was the most used tool to evaluate vision-related QoL (VRQoL) being used in 16 studies. Contact lens wear was the most common non-surgical intervention in 13 studies showing improvements in certain domains. The assessment of VRQoL across the studies revealed moderate to significant impairments in several key QoL domains including emotional, social and economic well-being. Patients with keratoconus who have not undergone surgery experienced significant impact on both physical and psychological dimensions. While contact lenses can improve visual function especially in the early stages of the disease but had limitations.

## Introduction

Keratoconus (KCN) is an irreversible bilateral chronic disease that affects the cornea leading to progressively reduced quality of life which is usually seen during puberty and progressing till the third or fourth decade [1–7]. The disease results in thinning and protrusion of the cornea as well as reduction in visual acuity due to irregular astigmatism, high-order aberrations, and scarring. There is the risk of progression to permanent corneal damage including blindness [1, 3, 4]. Risk factors that associated with keratoconus include age younger than 30 years old [1, 8, 9], the mechanical movement of eye rubbing and family history [1, 5, 8, 9]. Management of the condition depends on preventing progression and improving visual acuity [1, 8]. Non-surgical management includes visual aids such as glasses, contact lenses including scleral lenses maybe utilised to improve vision. Surgery may be used to prevent progression such as with cross-linking or improve vision such as with intrastromal ring segments and corneal transplantation [1, 8]. The choice of how to improve vision in keratoconus depends on the corneal parameters, patients factors along with the costs of treatment as well as available resources [1, 2].

Keratoconus significantly impacts patients’ QoL, compared with other ocular conditions such as macular degeneration, due to the chronic visual disability it imposes [6, 10]. Vision-related quality of life (VR-QoL) assessments using patient-reported outcome measures (PROMs) provide valuable insights into the subjective impact of keratoconus on a patient’s life [6, 10–12]. Commonly used surveys, such as the National Eye Institute Visual Function Questionnaire (NEI VFQ) and the Short-Form 36 (SF-36), have limitations due to their lack of specificity for keratoconus [10, 13, 14]. Tools such as Keratoconus Outcomes Research Questionnaire (KORQ), designed specifically for patients with the disease, uniquely capture the keratoconus-specific QoL challenges and are the preferred tool for evaluating the impact of the disease on daily functioning [11, 13]. Understanding the impacts on QoL of the range of treatments for keratoconus can inform clinicians on treatment priorities and patients in deciding on treatment pathways as well as inform health policy.

This study aimed to systematically evaluate the QoL in keratoconus patients who had not undergone surgical intervention, focusing on those managed with glasses, contact lenses, both or no treatment. By addressing the limitations of previous studies, we intended to provide a clearer understanding of the visual and psychosocial impacts of keratoconus in non-surgically managed patients, utilising validated assessment tools.

## Methods

**Fig. 1:**
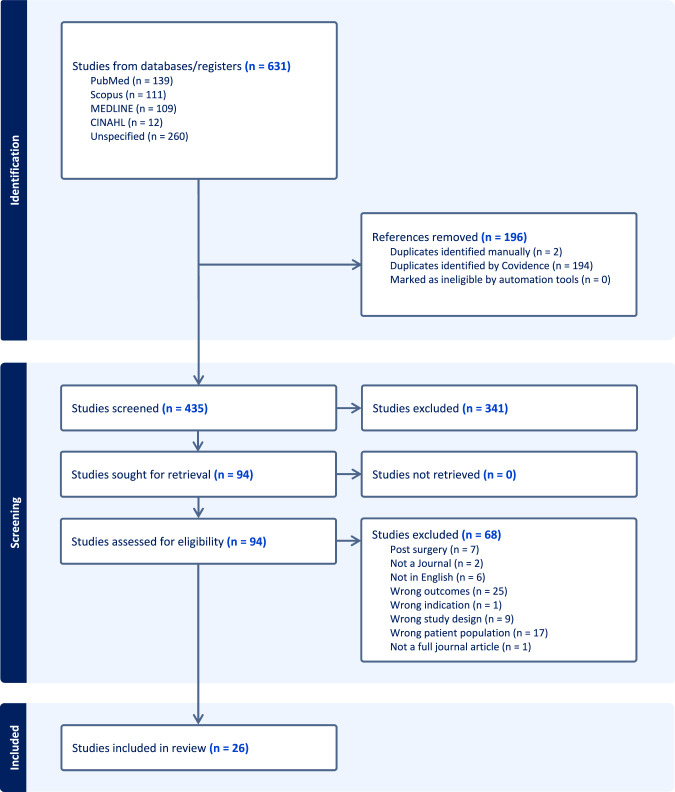
PRISMA flow chart for the Systematic review on QoL in patients with keratoconus managed non-surgically.

A search strategy was designed using 5 different search databases: PubMed, Scopus, Medline, Emcare and CINAHL (Supplemental data 1). No restrictions or filters were added in the initial search to prevent the possibility of missing important articles then a filter was added limiting to human(s). A Grey literature search was conducted via OpenGrey for more comprehensive search. The search yield was conducted using the PRISMA framework (Fig. 1) and achieved in 3 stages. In the initial stage, 2 separate reviewers (DS, RK) screened the titles and abstracts against the inclusion and exclusion criteria to determine suitability. Inclusion criteria were studies assessing the quality of life of patients with keratoconus who have not had a surgical intervention to manage their keratoconus. Studies that have not assessed quality of life in patients with other ocular diseases or with keratoconus that have been managed with any surgical intervention (such as cross-linking, rings, corneal transplantation and laser) were excluded. Additionally, articles written in English (or questionnaires translated into English by the authors) were only included to prevent any translation errors. Articles were classified as (1) definitely relevant (2) possibly relevant, or (3) definitely not relevant. For any discrepancy, a third reviewer (HK) was consulted for further assistance. Any articles with unclear suitability were thoroughly reviewed by a third reviewer (HK). Articles written in languages other than English were excluded. Data was then uploaded on Covidence and Excel (Microsoft 365) was used for data extraction. Questionnaires that focused on keratoconus were assessed for their quality and used to determine different aspects of QoL.

### Data extraction

Data extracted from the included studies were number and age of participants, keratoconus severity, use of non-surgical interventions such as glasses and/or contact lenses, type of questionnaire used to assess QoL and the VRQoL domains used in those questionnaires as well as the outcomes of the studies. Data on the sub-domains of VRQoL were also extracted, such as in NEI-VFQ questionnaires; general health, general vision (GV), ocular pain (OP), near activities, distance activities (DA), vision-specific social function, vision-specific mental health, vision-specific role difficulties, vision-specific dependency, driving, colour vision, peripheral vision and overall assessment.

### Bias assessment

Quality of the questionnaires was determined by psychometric properties, validity, reliability, and responsiveness [13]. The appropriate tools for the type of study were used to evaluate bias. These included the randomised controlled trials Cochrane RoB2.0 Tool [15] which enabled evaluation of subject selection, performance, detection, attrition and reporting. Non-randomised studies of interventions were assessed with the ROBINS-I tool [16]. The ROBINS-I tool Bias evaluated selection, comparability and exposure in the non-randomised studies. In addition to above, the quality of the articles was assessed using the criteria developed by Kandel et al. [13].

## Results

Six hundred and thirty-one articles were retrieved from the five databases and uploaded to Covidence. A total of 196 articles were duplicates and removed. A total of 435 were then screened for suitability. Three hundred and forty-one articles were deemed irrelevant. A total of 70 articles were screened based on the inclusion and exclusion criteria. A total of 26 articles were included for data extraction. No further articles were included after review of the reference lists of the included articles. The Grey articles search did not reveal any results (Fig. 1)

### Included studies

The studies included a mix of observational cohort studies (*n* = 3), cross sectional studies (*n* = 13), observational studies (*n* = 4), comparative studies (n = 2), a retrospective study (*n* = 1), interventional studies (*n* = 2) and a randomised clinical trial (*n* = 1). Out of the 26 articles, 3 articles used control groups while the rest did not. The primary focus of all the studies was to assess the QoL in patients diagnosed with keratoconus who had not undergone surgical interventions. Studies had sample sizes ranging from 25 to 1209 participants. The NEI VFQ-25 was the most commonly used tool to evaluate vision-related QoL (VRQoL) used in 16 studies. Some studies used other tools such as the KORQ (*n* = 3 studies), Contact Lens Impact on Quality of Life (CLIQ) (*n* = 1) and Vision and Quality of Life Index (VisQoL; *n* = 1) (Table 1). Five papers combined 2 different questionnaires in their study. Articles explored and compared glasses, contact lenses, both or none (being uncorrected) with varying percentages across the studies (Table 2). Contact lens wear was the most common non-surgical intervention in 13 studies. Five studies evaluated contact lenses in a range of grades of keratoconus including early-stage disease.

**Table 1:** Details of the questionnaires used to assess QoL in studies on patients with keratoconus managed non-surgically.

Questionnaire	Number of questions	Number of subscales	Number of articles (%)
NEI VFQ	25–39	11–12	16 (61.5)
KORQ	29	2	3 (11.5%)
IVI	28–32		2 (7.7%)
CLIQ	28		1 (3.9%)
VisQoL	6		1 (3.9%)
Tomey KC screening system	12		1 (3.9%)
History and contact lens performance questionnaire	3		1 (3.9%)
CVAQC	NS		1 (3.9%)
The Refractive Status and Vision Profile	6		1 (3.9%)
Zung SDS questionnaires	20		1 (3.9%)
PHQ-9	9		1 (3.9%)
CHU9D	9		1 (3.9%)

*CVAQC* Cardiff Visual Ability Questionnaire for children, *CHU9D* generic paediatric health outcome, *CLIQ* contact lens impact on quality of life, *IVI* impact of vision impairment, *KORQ* Keratoconus Outcomes Research Questionnaire, *NEI VFQ-25* National Eye Institute Visual Functioning Questionnaire 25, *NS* not stated, *PHQ-9* patient health questionnaire, *VisQoL* vision and quality of life index.

**Table 2:** The percentage of keratoconus patients who were uncorrected, wearing contact lenses or spectacles in publications reporting the use of different types of non-surgical interventions with QoL assessments.

Study	Uncorrected	Spectacles	Contact lenses	Combined
Larkin et al. [27]		57%	3%	7%
Kreps et al. [19]	18%	49.4%	32.5%	
Dudeja et al. [30]		58.5%	41.4%	
Gothwal et al. [21]		21%	33%	4%
Gothwal et al. [26]	21%	21%	51%	21%
Kandel et al. [11]	31.4%	62.9%	5.7%	
Al Zabadi et al. [38]	47.6%	44%	100%	

Keratoconus severity was included in some studies and was graded into five groups in 12 of the articles (46.1%). The severity varied from mild (Grade I), moderate (Grade II), severe (Grade III), very severe (Grade IV) and extremely severe (Grade V) (Table 3). Table 3 also presents the country, gender, ethnicity, education status and employment of patients in the included studies.

**Table 3:** Keratoconus severity in percentages ranging from mild (grade 1) to extremely severe (grade V) as well the year, country, ethnicity, age in range of years and mean with standard deviation, gender, education and employment status from studies reporting on keratoconic patients with non-surgical management and assessment of QoL.

Study	Year	Country	Ethnicity	Sample size	Male	Age mean ± STD	Age range	Education	Unemployment	Grade of keratoconus				
										Mild I	Moderate II	Severe III	Very severe IV	Extremely severe V
Gothwal et al. [26]	2013	India	Indians	160	63%	23.3 ± 5.8	18–53	NS	69%	0%	37%	63%	0%	0%
Kurna et al. [33]	2014	Turkey	Turkish	60	47%	29.36 ± 10.60	NS	100%	NS	13.3%	46.6%	20%	20%	0%
Mahdaviazad et al. [28]	2018	Iran	Iranian	125	55%	NS	14–57	100%	NS	4.5%	71.2%	14.4%	0%	0%
Panthier et al. [32]	2020	France	Caucasian and others	101	67.3%	28.04 ± 9.30	15–57	NS	NS	4.9%	13.9%	70.3%	0%	0%
Chan et al. [39, 40]	2020	Australia	Multiple	104	57%	31	23–44	NS	34.6%	5%	25%	29%	0%	0%
Kreps et al. [19]	2021	Belgium	NS	118	64%	NS	18–67	NS	NS	23.6%	37.1%	25.8%	11.2%	2%
Baudin et al. [17]	2021	France	French	27	62%	33.5 ± 13.8	NS	NS	NS	20%	39%	24%	17%	0%
Dudeja et al. [30]	2021	India	Indians	328	64.6%	NS	12–36	NS	NS	24.4%	47.6%	3.6%	23.2%	0%
Kandel et al. [6]	2022	Australia and 5 other countries	Caucasians/ white and Aust	1557	50.8%	NS	11–100	NS	NS	30%	38.8%	25.1%	0%	0%
Gothwal et al. [21]	2022	India	Indians	574	57%	NS	18–40	82%	61%	53%	28%	4%	15%	0%
Schummer et al. [36]	2023	Belgium	NS	154	57.1%	35.0 ± 10.6	NS	NS	NS	53.2%	28.6%	13.6%	2.6%	0%
Kandel et al. [11]	2023	Australia	White and non-white	542	67.7%	31.8 ± 12.5	NS	NS	NS	26.3%	45%	28.7%	0%	0%

*STD* Standard Deviation, *NS* Not Stated.

### NEI-VFQ

The NEI-VFQ tool was used in the majority of the studies (*n* = 16, 61.5%) and was translated in different languages including English (*n* = 1), Hindi (*n* = 1), Chinese (*n* = 1), French (*n* = 2), Dutch (*n* = 2), Spanish (n1), Arabic (*n* = 1), Turkish (*n* = 3) and Farsi (*n* = 2). Studies used 11 (*n* = 6/16) or 12 (*n* = 10/16) subscales of the NEI-VFQ to assess QoL of patients with keratoconus. The assessment of VRQoL across the studies revealed moderate to significant impairments in several key domains. General vision mean scores ranged from 29.4 [17] to 88.2 [18], reflecting a range of vision impairments across the studies. Impairments in near activities mean (4.07 [19] to 91.6 [18]) and distance activities (3.45 [17], 93.4 [18]) were consistently reported depending on the specific study. Overall QoL scores varied across studies and some studies did not include the overall score from the NEI-VFQ (Supplemental data 2).

The use of contact lenses (rigid gas-permeable, hybrid, or soft lenses) across the studies using NEI- VFQ tool showed improvements in certain domains, particularly in distance activities and general vision. Non-surgical interventions such as wearing contact lenses or spectacles, did not fully restore normal QoL levels. Patients reported persistent discomfort with contact lens wear, such as ocular pain and dryness, which continued to negatively impact their daily activities and overall well-being.

### KORQ

Three studies used the KORQ (*n* = 3, 11.5%) and utilised between 18 and 29 questions of the KORQ. The Questionnaire is divided into a symptomatic score with 18 subscales and visual function score with 11 subscales [20] (Table 4). One study evaluated the difference between the better and the worse eye using the different subscales of the KORQ [21] (Table 4). Gothwal et al. [21] explored the impact of keratoconus on patient’s daily activities and symptoms. The study presented that individual with worse visual acuity and higher ocular aberrations experienced greater activity limitations [21]. In addition, the study reported that females and employees had significant symptoms compared to men and non-employed individuals, respectively [21]. Interestingly, the study showed that even mild keratoconus had a noticeable impact on QoL, suggesting that patients across all disease severities require attention [21]. In another study by Kandel et al. [11], female patients had also low score when it came to symptoms as well as in activity limitation [11]. In this study, severe keratoconus group showed lower scores in the domains measured [11]. Al Bdour et al. [22] also showed females to have worse symptoms scores compared to men but it was not statistically significant [22].

**Table 4:** The Keratoconus Outcomes Research Questionnaire (KORQ) scores with the 2 main subscales of Activity limitation score mean with standard deviation and Symptoms mean with standard deviation in keratoconic patients with non-surgical management and QoL assessment Key: G: Grade of Keratoconus severity.

Study	Activity limitations score mean		Symptoms score mean	
Gothwal et al. [21]	Better eye	Worse eye	Better eye	Worse eye
	G1 0.90 ± 1.48	1.07 ± 1.70	0.47 ± 1.27	0.71 ± 1.32
	G2 0.94 ± 1.43	0.86 ± 1.41	0.20 ± 1.28	0.30 ± 1.20
	G3 0.66 ± 1.17	1.18 ± 1.21	0.31 ± 1.28	0.27 ± 1.29
	G4 0.77 ± 1.63	0.75 ± 1.47	0.67 ± 1.44	0.26 ± 1.36
Kandel et al. [11]	Mild	60.8		50.9
	Moderate	58.1		55.8
	Severe	52.5		56.6
Al Badour et al. [22]	58.28		72.95	

### Other questionnaires

Other questionnaires were used in 4 articles. Five articles combined two questionnaires for more comprehensive evaluation. These included the History and Contact Lens Performance questionnaire, Generic paediatric Health outcome (CHU9D) and Cardiff Visual questionnaire for children (Table 1).

### Psychometric properties

Validity, reliability and responsiveness were the main attributes to assess the quality of the tools used to evaluated QoL in the included studies [13]. The majority of the articles did not report the psychometric properties of the questionnaire used to assess QoL which made it difficult to assess these properties. For the 3 articles which mentioned psychometric measurements Rasch analysis was utilised. In the study that was performed by Gothwal et al. [21], psychometric properties of the KORQ were measured through Rasch analysis and confirmed to be robust. Subscales of Activity Limitations and Symptoms demonstrated adequate response, consistency and reliability across the different groups. Kandel et al. [11] showed that Activity limitation (AL) and Symptoms (SY) scales of the KORQ had also robust psychometric properties including well-functioning response categories, unidimensionality, excellent measurement precision, and satisfactory fit statistics [11]. Erdurmus et al. [23] also validated the 28-item CLIQ Questionnaire using Rasch analysis with psychometric analysis showing that it is a valid and reliable measure of QoL in keratoconus patients wearing contact lenses [23].

### Bias assessment

The risk of bias assessment revealed variability across the included studies. Risk of bias was determined as low for 3 studies, moderate for 14 studies, moderate to high in 6 studies and 1 study had high to serious risk. For the 14 studies that had moderate risk of bias, this was primarily due to confounding, selection bias, and reliance on subjective self-reported outcomes. Thirteen studies used cross-sectional designs frequently lacked adjustment for key confounders such as socioeconomic factors, while single-centre recruitment limited the generalisability of findings. Notably, studies like Steinberg et al. [24] and Wu et al. [25] displayed moderate-serious or high risk of bias due to incomplete follow-up, unmeasured confounders, and subjective measures and undermining the robustness of their conclusions. Three studies, including Gothwal et al. [26] and Larkin et al. [27], were assessed as having a low risk of bias, benefiting from rigorous methodology and randomisation (Supplemental data 3).

## Discussion

This systematic review highlighted the significant impact keratoconus has on the QoL in patients who have not undergone surgical interventions. Twenty-six articles were included and systematically reviewed. Different tools were used by the studies to assess QoL with the majority of the studies using the NEI-VFQ tool with 11 or 12 subscales. The KORQ was second in frequency of use by the studies; 3 studies utilised the tool for their assessment using the 2 main subscales. Other studies utilised other tools with some using 2 different tools for a more comprehensive assessment of QoL.

A significant portion (61.5%) of the reviewed studies utilised the NEI VFQ to measure QoL. This tool has proven useful in assessing general visual function and wellbeing but has limitations in capturing the keratoconus-specific challenges patients face, such as irregular astigmatism, higher-order aberrations, and glare sensitivity [13]. As keratoconus onsets in the young and is a progressive disease with more specific symptoms resulting from myopia and irregular astigmatism compared to many other eye diseases, it is important to have more specific tools to determine QoL [21]. Despite the NEI-VFQ tool not being specific for keratoconus, it helped patients report different aspects of daily life such as dependency and social isolation as their condition progressed. The NEI-VFQ was also able to report on advanced cases of keratoconus where non-surgical options such as glasses or contact lenses failed to provide adequate relief [28]. Additionally, across the studies reviewed, there was clear evidence of visual function impairment, which strongly affected a patient’s ability to perform daily tasks. Both near and distance vision were notably compromised, along with consistent reports of difficulties in routine activities such as reading and driving. The progressive nature of keratoconus, even in patients managed non-surgically, contributed to a steady decline in visual acuity and functional independence, thereby affecting their overall well-being [29, 30].

Three studies incorporated the KORQ; which is disease specific and psychometrically robust for keratoconus [21]. The KORQ provided a more detailed account of the specific difficulties encountered by keratoconus patients, with higher sensitivity to vision-related and ocular comfort symptoms and activity limitations [21].

Contact lens wear was the most common non-surgical intervention reported and was evaluated in a range of grades of keratoconus including in early-stage disease. In studies such as in Kreps et al. [19], they explored a variety of hybrid and special fitted lenses such as rigid gas-permeable, scleral and hybrid lenses prescribed to improve visual acuity [19]. Different lenses were trialled on all keratoconus patients despite the severity of the disease and in some studies, the researchers switched patients to use different lenses during the study [19, 31]. However, despite the visual benefits provided by different types of lenses, patients frequently reported persistent discomfort. Symptoms such as ocular dryness, pain, discomfort, lens fogginess from debris and the physical discomfort associated with prolonged lens use were commonly mentioned [17, 19, 31]. Even with proper lens fitting, these issues reduced patients QoL and caused significant daily challenges. This highlights the inadequacies of contact lenses in fully addressing both the visual and symptomatic burdens of keratoconus [18, 19, 31].

The severity of keratoconus played a role in determining the extent of QoL impairment in patients who had not had surgical interventions. Patients with moderate to severe keratoconus in the study by Panthier et al. [32] reported more pronounced reductions in visual function, which was directly reflected in the included patients QoL scores [32]. Studies categorising patients by disease severity demonstrated that those with advanced keratoconus experienced worse functional outcomes [33]. The psychological toll of living with a progressively worsening condition was reflected in lower mental health scores in QoL assessments [32]. Increased anxiety and stress especially in younger patients, indicating the pervasive impact keratoconus has not only on vision but on overall mental well-being [32, 34].

The assessment of risk of bias in the included studies revealed notable strengths and weaknesses of the studies that have important implications for interpreting the findings of this systematic review. While a randomised controlled trial by Larkin et al. [27] demonstrated a low risk of bias, the majority of studies were classified as having moderate to serious risks of bias. These risks were largely attributed to methodological limitations such as selection bias and inadequate adjustment for confounding variables [21, 23, 31, 34]. Such methodological weaknesses limited the robustness and generalisability of the findings and highlighted the challenges in deriving definitive conclusions about the QoL in patients with keratoconus. For instance, a common limitation among cross-sectional and observational studies was the inability to establish relationships between keratoconus severity and quality of life outcomes, as well as the absence of consistent adjustment for key socioeconomic and psychological factors known to influence patient-reported outcomes [11, 35, 36]. These gaps underscore the need for cautious interpretation of the reported associations. The reliance on single-centre recruitment such as in Gothwal et al. [21] and Mahdaviazad et al. [28] as well as small sample sizes such as in Kurna et al. [33] in further restricted the generalisability of the findings. Additionally, the lack of blinding and randomisation in many comparative studies introduced further risks of bias, which need to be considered when interpreting these results [31, 37]. One study by Steinberg et al. [24] showed high risk of bias as the control group was mixed with myopic patients and the number was not equal to the keratoconus group [24]. The study eliminated the progressive group which could potentially reflect on the results obtained [24]. It should also be noted that we did not compare the QoL of non-surgical vs surgical management of keratoconus. Such that the findings of our review cannot be used to determine which management may be optimal for a particular patient group.

## Conclusion

Keratoconus patients managed non-surgically report considerable declines in quality of life with impacts on both physical and psychological dimensions. While contact lenses can improve visual function especially in the early stages of the disease, there are issues related to discomfort and functional limitations experienced by patients. Prospective, comparative studies with keratoconus-specific measures to assess patient-reported outcomes are needed to further understand QoL impacts in keratoconus.

Supplemental material is available at *Eye’s* website.

## Supplementary information

Supplemental data 1

Supplemental data 2

Supplemental data 3

## Data Availability

All data analysed in this systematic review are publicly available in the articles listed in the reference section. No new data were generated. Supplementary materials, including data extraction tables and PRISMA checklists, are included within the article.
