# Supplementary material for: What is the quality-of-life status of patients with keratoconus who have not had a surgical intervention? A systematic review
Source: Eye (Lond). 2025 Oct 22;39(18):3229–36. doi: 10.1038/s41433-025-04053-0 (PMC12669613; doi:10.1038/s41433-025-04053-0)
Supplement: Supplementary file 1 — Supplemental data 1 [file 41433_2025_4053_MOESM1_ESM.docx]

# Literature/systematic review worksheet

**Requester:** Daliya Sari (dsar0922@uni.sydney.edu.au)

**Search description:** Research question

“Can quality of life of patients with keratoconus improve without surgical intervention?".

**Keywords/concepts:**

1. Keratoconus

OR Keratoconic OR keratocon OR “corneal ectasia” OR “cornea ectatic” OR Corneal thinning OR corneal degeneration.

2. “Quality of life”

OR "Activities of Daily Living" OR "Activity limitation" OR "daily life activity" OR "life Satisfaction" or "physical quality of life" OR "Self esteem" OR "Functional Status" OR driving OR (limit* W2 activit*) OR (quality W2 life) OR (vision W2 disab*) OR (vision W2 quality) OR "Psychological Well-Being" OR Psychological OR psychosocial OR Wellbeing OR well-being OR "Mental Health" OR "Depression" OR "Anxiety" OR Emotion* OR "Health Status" OR "Body Image" OR "Body Dissatisfaction" OR "Psychosocial Functioning" OR "Social Participation" OR "Self Concept" OR social OR "social wellbeing" OR "Social Interaction" OR "Social Isolation" OR "Social Stigma" OR "Resilience, Psychological" OR "Stress, Psychological" OR "Psychosocial Deprivation" OR symptom* OR glare OR photosensitivity OR Photophobia OR Economic OR Cost OR "patient outcome" OR "Patient?reported outcome*"

3. "Patient reported outcome measures"

OR PROM OR questionnaire OR question* OR qualitative OR interviews OR "focus group discussion".

**Inclusion criteria:** On keratoconus, humans, pre surgical, surgically naïve, glasses, lenses, contact lenses, no intervention.

**Exclusion criteria:** Post surgery OR “cross-link” OR “corneal transplant” OR laser OR UV laser,Non-human.

**Date limitation:** Non.

**Population:** Patient with keratoconus who are surgically naïve and have an assessment of their quality of life

**Databases:** Medline, Emcare, CINAHL, Scopus and PubMed

**Methodology for Databases**

**OVID Databases**

| **MEDLINE(R) including Daily update <1996-current>** | | |
| --- | --- | --- |
| **Search Strategy** | **Search link** | **Notes** |
| **1**  Keratoconus/ keratocon.mp. (6884)  **2**  Keratoconic.mp. (1051)  **3**  "corneal ectasia" OR “cornea ectatic”.mp. (861)  **4**  "Corneal thinning".mp. (669)  **5**  "corneal degeneration".mp. (319)  **6**  1 OR 2 OR 3 OR 4 OR 5 (8308)  **7**  "Quality of Life"/(281082) **8**  "Activities of Daily Living ti,ab" OR "Activity limitation" OR "daily life activity" OR "life Satisfaction" OR "physical quality of life").mp.(14567) **9**  ((limit* adj2 activit*) OR (quality adj2 life) OR (vision adj2 disab*) OR (vision adj2 quality) OR driving OR "Functional Status").mp. (651225)  **10**  (Emotion* OR Psychological OR psychosocial OR Wellbeing or well-being).mp. OR "Psychological Well-Being".ti,ab. or "Resilience, Psychological".mp. OR "Stress, Psychological".mp. OR "Psychosocial Deprivation".mp. OR "Mental Health".ti,ab. OR "Depression".mp. OR "Anxiety".mp. OR "Health Status".mp. OR "Psychosocial Functioning".mp. OR "Self Concept".mp. OR "Body Image".mp. OR "Body Dissatisfaction".mp. OR social.mp. OR "Social Participation".mp. OR "social wellbeing".mp. OR "Social Interaction".mp. OR "Social Isolation".mp. OR "Social Stigma".mp. OR symptom*.ti,ab. OR glare.mp. OR photosensitivity.mp. OR Photophobia.mp. OR Economic.mp. OR Cost.mp. OR "patient outcome".ti,ab. OR "Patient?reported outcome*".mp.(4357501)  **11** 7 OR 8 OR 9 OR 10 (4692628)   \|  \| \| --- \|   **12** ("patient reported outcome measures" OR PROM OR questionnaire OR question* OR qualitative OR interviews OR "focus group discussion").mp. (1940057)  **13** 6 AND 11 AND 12 (109)   \|  \| \| --- \|  \|  \| \| --- \| |  | 109 |
| **Emcare** |  |  |
| \| **1**  Keratoconus.mp. OR keratoconus/ or keratocon.mp (1657)  **2**  Keratoconic.mp. (231)  **3**  "corneal ectasia" OR “cornea ectatic”.mp. (189)  **4**  "Corneal thinning".mp. (129)  **5**  "corneal degeneration".mp (36)  **6**  1 OR 2 OR 3 OR 4 OR 5 (1803)  **7**  "quality of life"/ (146161)  **8**  "Activity limitation" OR "daily life activity" OR "life Satisfaction" or "physical quality of life").mp. (47828)  **9** "Activities of Daily Living".ti,ab. OR (limit* adj2 activit*).mp. OR (quality adj2 life).mp. OR (vision adj2 disab*).mp. OR (vision adj2 quality).mp. OR driving.mp. OR "Functional Status"/ OR "Psychological Well-Being".mp. OR "Self esteem".mp. (346561)  **10** (Emotion* OR Psychological OR psychosocial OR Wellbeing or well-being).mp. OR "Psychological Well-Being".ti,ab. OR "Resilience, Psychological".mp. OR "Stress, Psychological".mp. OR "Psychosocial Deprivation".mp. OR "Mental Health".ti,ab. OR "Depression".mp. OR "Anxiety".mp. OR "Health Status".mp. OR "Psychosocial Functioning".mp. OR "Self Concept".mp. OR "Body Image".mp. OR "Body Dissatisfaction".mp. OR social.mp. OR "Social Participation".mp. OR "social wellbeing".mp. OR "Social Interaction".mp. OR "Social Isolation".mp. OR "Social Stigma".mp. OR symptom*.ti,ab. OR glare.mp. OR photosensitivity.mp. OR Photophobia.mp. OR Economic.mp. OR Cost.mp. OR "patient outcome"/ OR "Patient?reported outcome*".mp. (1895683)  **11** 7 OR 8 OR 9 OR 10 (2046972)  **12** "patient reported outcome measures" OR PROM OR questionnaire OR question* OR qualitative OR interviews OR "focus group discussion").mp. (939218)  **13** 6 AND 11 AND 12 (40) \|  \|  \| \| --- \| --- \| --- \| |  | 40 |
| **CINAHL** | | |
| S1 MM "Keratoconus" (823)  S2 Keratoconus OR keratocon OR Keratoconic OR "corneal ectasia" OR "corneal thinning" OR "corneal degeneration" OR ectasia OR cornea ectatic (653)  S3 MM "Quality of Life" (67,465)  S4 MM "Psychological Well-Being" (18019)  S5 MM "Activities of Daily Living" OR "daily life activity"  (12,076)  S6 "physical quality of life" OR "social wellbeing" OR "daily life activity" OR "Activities of Daily Living" OR quality N2 life OR "vision related quality of life" OR vision N2 quality OR Wellbeing OR well-being OR "life Satisfaction" OR "Self esteem" OR Emotion* OR social OR Psychological OR psychosocial OR "Activity limitation" OR limit* N2 activit* OR vision N2 disab* OR “Mental Health” OR “Depression” OR “Anxiety” OR "Health Status" OR "Psychosocial Functioning" OR "Self Concept" OR "Body Image" OR "Body Dissatisfaction" OR social OR "Social Participation" OR "social wellbeing" OR "Social Interaction" OR "Social Isolation" OR "Social Stigma" (1,597840)  S7 AB symptom* OR glare OR photosensitivity OR Photophobia OR Economic OR Cost OR “patient outcome” OR “Patient?reported outcome*”  (853639)  S8 ("patient reported outcome measures" OR PROM or questionnaire OR question(s) OR qualitative OR interviews OR "focus group discussion")  (983691)  S9 S1 OR S2  (1317)  S10 S3 OR S4 OR S5 OR S6 OR S7  (2154239)  S11 S8 AND S9 AND S10  (12) |  | 12 |
| **Scopus (via your institution)** | | |
| TITLE-ABS-KEY ( "keratoconus" ) OR ALL ( keratoconic OR keratocon OR "corneal ectasia" OR "corneal thinning" OR "corneal degeneration" ) AND TITLE-ABS-KEY ( "quality of life" ) OR TITLE-ABS-KEY ( "Activities of Daily Living" OR "Activity limitation" OR "daily life activity" OR "life Satisfaction" OR "physical quality of life" ) OR ALL ( ( quality AND w2 AND life ) OR ( limit* AND w2 AND activit* ) OR ( vision AND w2 AND disab* ) OR ( vision AND w2 AND quality )) TITLE-ABS-KEY ( "Functional Status" OR driving OR "Self esteem" OR emotion* OR psychological OR psychosocial OR wellbeing OR well-being OR "Resilience, Psychological" OR "Stress, Psychological" OR "Psychosocial Deprivation" OR "Mental Health" OR "Depression" OR "Anxiety" OR "Health Status" OR "Psychosocial Functioning" OR "Self Concept" OR "Body Image" OR "Body Dissatisfaction" ) OR ALL ( social OR "Social Participation" OR "social wellbeing" OR "Social Interaction" OR "Social Isolation" OR "Social Stigma" ) OR TITLE-ABS-KEY ( symptom* ) OR ALL ( glare OR photosensitivity OR photophobia OR economic OR cost OR "patient outcome" OR "Patient?reported outcome*" ) AND ALL ( "patient reported outcome measures" OR prom OR questionnaire OR question OR qualitative OR interviews OR "focus group discussion" ) AND ( LIMIT-TO ( EXACTKEYWORD , "Human" ) OR LIMIT-TO ( EXACTKEYWORD , "Humans" ) ) |  | Limit to humans  111 |
| **PubMed** | | |
| (((("Keratoconus"[Mesh] OR Keratoconus OR Keratoconic OR keratocon OR "corneal ectasia" OR "cornea ectatic" OR "corneal thinning" OR "corneal degeneration")) AND (("quality of life"[tiab] OR "Activities of Daily Living" [Mesh] OR "Activity limitation" OR "daily life activity" OR "life Satisfaction" or "physical quality of life" OR "Functional Status"[Mesh] OR driving OR (limit* W2 activit*) OR (quality W2 life) OR (vision W2 disab*) OR (vision W2 quality) OR "Psychological Well-Being" OR Psychological OR psychosocial OR Wellbeing OR well-being OR "Resilience, Psychological"[Mesh] OR "Stress, Psychological"[Mesh] OR "Mental Health" [Mesh] OR "Depression" OR "Anxiety" OR Emotion* OR "Health Status"[Mesh] OR "Psychosocial Functioning"[Mesh] OR social OR "social wellbeing" OR "Social Participation"[Mesh] OR "Social Interaction"[Mesh] OR "Social Isolation"[Mesh] OR "Social Stigma"[Mesh] OR "Psychosocial Deprivation"[Mesh] OR "Self esteem" OR "Self Concept"[Mesh] OR "Body Image"[Mesh] OR "Body Dissatisfaction"[Mesh] OR symptom*[tiab] OR glare OR photosensitivity OR Photophobia OR Economic OR Cost OR "patient outcome" OR "Patient?reported outcome*" ))) AND (" patient reported outcome measures" OR PROM OR questionnaire OR question* OR qualitative OR interviews OR "focus group discussion") |  | 139 |
