# Supplementary material for: What is the quality-of-life status of patients with keratoconus who have not had a surgical intervention? A systematic review
Source: Eye (Lond). 2025 Oct 22;39(18):3229–36. doi: 10.1038/s41433-025-04053-0 (PMC12669613; doi:10.1038/s41433-025-04053-0)
Supplement: Supplementary file 2 — Supplemental data 2 [file 41433_2025_4053_MOESM2_ESM.docx]

| **Supplemental data 2**: Publications reporting VRQoL Domain Scores by NEI VFQ-25 Tool using mean and standard deviation of patient’s number scores for keratoconic patients with non-surgical management and assessment of QoL. Key: Spec: spectacles, CL: contact lenses, NS Not stated. |
| --- |
| \| **Study** \| **General Vision Score** \| **Ocular Pain Score** \| **Near Activities Score** \| \| **Distance Activities Score** \| **Social function** \| **Mental Health** \| **Role difficulty** \| **Dependency** \| **Driving** \| \| **Colour**  **vision** \| **Peripheral vision** \| **Overall QoL Score** \| \| --- \| --- \| --- \| --- \| --- \| --- \| --- \| --- \| --- \| --- \| --- \| --- \| --- \| --- \| --- \| \| Panthier et al ^32^ \| 64.1 ± 18.6 \| 71.5 ± 25.5 \| 76.6 ± 21.6 \| \| 78.1 ± 22.9 \| 89.1 ± 20.2 \| 62.2 ± 24.5 \| 71.2 ± 22.8 \| 83.0 ± 25.0 \| NS \| \| 97.0 ± 11.4 \| 81.0 ± 23.1 \| 77.3 ± 17.8 \| \| Baudin et al ^17^ \| 29.4±23.4 \| 17.1±30.9 \| 28.5±27.9 \| \| 15.6±26.2 \| 9.9±20.6 \| 21.2±26.0 \| 20.8±28.6 \| 11.0±41.8 \| 19.1±24.8 \| \| 2.6±7.9 \| 23.7+/-28.2 \| 19.5±19.1 \| \| Dudeja et al ^30^ \| 71.91±28.3 \| 80±21.7 \| 82.82±14.7 \| \| 78.8±17.7 \| 88.12±15.7 \| 62.9±18.5 \| 81.9±21.6 \| 92.1±14.5 \| \| 83.3±11.6 \| 96.6±8.6 \| 94.1±6.6 \| NS \| \| Kurna et al ^33^ \| 65.0 ± 20.6 \| 54.0 ± 23.8 \| \| 76.0 ± 23.0 \| 84.0 ± 18.0 \| 85.0 ± 24.0 \| 67.0 ± 27.8 \| 77.2 ± 26.4 \| 84.7 ± 26.4 \| \| NS \| 91.0 ± 17.0 \| 80.7 ± 17.4 \| 75.2 ± 17.2 \| \| Mahdaviazad et al ^28^ \| 65.7±19.2 \| 65.3±23.5 \| \| 75.2±21.3 \| 76.9±19.6 \| 79.6±22.9 \| 65.7±24.4 \| 68.9±26.8 \| 85.3±22.0 \| \| 77.4±22.7 \| 91.2±17.7 \| 81.6±22.1 \| 74.5±17.1 \| \| Al Zabadi et al ^38^ \| 69.6 ± 16.5 \| 66.5 ± 22.2 \| \| 77.3 ± 14.4 \| 72.3 ± 18.2 \| 80.7 ± 20.8 \| 56.4 ± 22.6 \| 58.5 ± 30.3 \| 80.0 ± 22.0 \| \| NS \| 96.0 ± 11.8 \| 92.0 ± 13.9 \| 75.84 ± 18.6 \| \| Kymes et al ^34^ \| 74.7 \| 75.6 \| \| 79.1 \| 78.4 \| 91.7 \| 74.9 \| 81.9 \| 94.4 \| \| 80.4 \| 96.9 \| 81.9 \| NS \| \| Ozcan et al^18^ \| 88.2 ± 10.5 \| 82.4 ± 12.3 \| \| 91.6 ± 9.2 \| 93.4 ± 9.8 \| 92.3 ± 8.6 \| 91.8 ± 7.9 \| 93.7 ± 8.2 \| 95.4 ± 7.8 \| \| NS \| 96.7 ± 6.3 \| 92.4 ± 7.7 \| 90.9 ± 7.3 \| \| Kreps et al ^19^ \| NS \| NS \| \| 4.07 ± 0.56 \| 3.45 ±0.22 \| NS \| NS \| 4.04 ± 0.64 \| NS \| \| NS \| NS \| NS \| NS \| \| Ortiz et al ^40^ \| Spec: 42.50 ± 21.52  CL: 72.00 ± 22.36 \| 75.00 ± 21.80  63.50 ± 27.46 \| \| 57.67 ± 27.94  81.33 ± 20.73 \| 51.66 ± 30.90  80.00 ± 18.79 \| 71.88 ± 29.32  89.00 ± 17.79 \| 54.33 ± 25.91  50.80 ± 19.40 \| 53.00 ± 38.74  75.00 ± 29.97 \| 68.40 ± 39.70  87.00 ± 25.00 \| \| 48.75 ± 31.68  79.76 ± 20.34 \| 83.33 ± 26.24  95.00 ± 10.21 \| 47.00 ± 31.27  82.00 ± 22.27 \| 58.31 ± 25.65  77.64 ± 16.51 \| \| Yildiz et al^41^ \| 43.4±18.1 \| 57.0±22.8 \| \| 54.1±21.2 \| 48.2±23.5 \| 64.8±22.1 \| 50.4±22.7 \| 50.5±23.8 \| 64.1±29.2 \| \| 39.2±37.4 \| 81.5±28.4 \| 63.0+/-24.8 \| 56.8+/-17.1 \|   Note: A number of studies ^24, 25, 31, 36, 37^ used NEI-VFQ but did not report scores for the different subscale. |
