# Supplementary material for: What is the quality-of-life status of patients with keratoconus who have not had a surgical intervention? A systematic review
Source: Eye (Lond). 2025 Oct 22;39(18):3229–36. doi: 10.1038/s41433-025-04053-0 (PMC12669613; doi:10.1038/s41433-025-04053-0)
Supplement: Supplementary file 3 — Supplemental data 3 [file 41433_2025_4053_MOESM3_ESM.docx]

**Supplemental data 3**: Type of studies, the risk of bias based on ROBINS – I and Rob 2 with reasons.

| **Study** | **Study Type** | **Bias Assessment Tool** | **Risk of Bias** | **Key Limitations** |
| --- | --- | --- | --- | --- |
| Gothwal et al ^43^ | Cohort Study | ROBINS-I | Low | Absence of control group, Limitations with literal responses |
| Kymes et al ^34^ | Cohort study | ROBINS-I | Moderate | Missing information such as keratoconus severity, socioeconomic data |
| Hashemi et al ^31^ | Comparative case series | ROBINS-I | Moderate | No randomisation or control group, inadequate adjustment for confounders, missing information such keratoconus severity and socioeconomic |
| Erdurmus et al ^23^ | Cross-Sectional Study | ROBINS-I | Moderate | No control group used, questions directed towards contact lenses use rather than QoL |
| Chan et al ^42^ | Cross-Sectional Study | ROBINS-I | Moderate | Inconsistent reporting of socioeconomic status |
| Larkin et al ^27^ | RCT | ROBIN -2 | Low |  |
| Ozcan et al ^18^ | Prospective Cross-Sectional Study | ROBINS-I | Moderate | Exclusion of some participants, lack of different skirt options in the fitted lenses, and limited sample size. |
| Panthier et al ^32^ | Prospective Cross-Sectional Study | ROBINS-I | Moderate | No control group; report only associations between potential causative factors and quality of life scores; a causativerelationship would require a longitudinal study |
| Gothwal et al ^21^ | Prospective Cross-Sectional Study/observational study | ROBINS-I | Moderate | Limited generalisability due to single-centre recruitment. Absence of proportional bias. Only explore activity limitation and symptoms. |
| Sahebjada et al ^35^ | Cross-Sectional Study | ROBINS-I | Moderate | Missing some data such as keratoconus severity and limited generalisability. |
| Schümmer et al ^36^ | Cross-Sectional Case-Control | ROBINS-I | Moderate | Single-centre recruitment, contrast sensitivity data for some patients as well as assessments, and missing data from the questionnaire. |
| Kreps et al ^19^ | Prospective Interventional Case Study | ROBINS-I | Moderate | Exclusion of certain patients, incomplete follow-up data, and short follow-up period. Lack of adjustment for possible confounding factors that can affect NEI-VFQ scoring and using a different version of the questionnaire. |
| Kandel et al ^11^ | Cross-Sectional Study | ROBINS-I | Moderate | Selection bias from certain clinics, Variability in information (Kmax use only). |
| Moschos et al ^44^ | retrospective Study | ROBINS-I | Low-Moderate | Questionnaires not specific to keratoconus |
| Al Bdour et al ^22^ | Cross-Sectional Study | ROBINS-I | Moderate | Selection bias due to recruitment criteria, no control group, length of the questionnaire and reliance on subjective self-reported outcomes. |
| Kandel et al ^6^ | Cross-Sectional Study | ROBINS-I | Moderate | Incomplete socioeconomic and no specific information in relation to use of non surgical interventions. |
| Baudin et al ^17^ | Prospective Observational | ROBINS-I | Moderate | Small group number and no control group, selection bias, missing data, significant drop out rate and short follow up time. |
| Steinberg et al ^24^ | Combined Prospective/Cross-Sectional | ROBINS-I | High-Serious | Reduced potential answers, one severity group was not used and lack of data correlation. |
| Wu et al ^25^ | Prospective Comparative Study | ROBINS-I | Moderate-Serious | Different lens designs were not studied, missing data, increased variability. |
| Dudeja et al ^30^ | Observational study | ROBINS-I | Moderate-Serious | Lack of control group, and missing long-term data. |
| Ortiz-Toquero et al ^40^ | Observational study | ROBINS-I | Moderate-Serious | Data variability, small size group, subjective measurement of outcomes and missing data. |
| Al Zabadi et al ^38^ | Cross-Sectional Study | ROBINS-I | Moderate-Serious | Small group size and did not specifically focus on exploring differences related to vision aids. |
| Yildiz et al ^41^ | Prospective Cohort Study | ROBINS-I | Moderate-Serious | Selection bias, small group and missing data. |
| Mahdaviazad et al ^28^ | Cross-Sectional Study | ROBINS-I | Moderate-Serious | Single-centre recruitment, selection bias, analysing questionnaire against demographics data and impact of treatment on questionnaire. |
| Kurna et al ^33^ | Observational Study | ROBINS-I | Moderate | Small sample size, missing data and single-centre recruitment. |
| Betts, G et al ^37^ | prospective interventional study | ROBINS-I | Moderate | Non-randomized design, lack of blinding, selection and subject bias, small group size and missing data. |

References:

40. Ortiz-Toquero S, Perez S, Rodriguez G, Juan V, Mayo-Iscar A, Martin R and De Juan V. The influence of the refractive correction on the vision-related quality of life in keratoconus patients. *Quality of Life Research* 2016; 25: 1043-1051. DOI: 10.1007/s11136-015-1117-1.

41. Yildiz E, Toklu M and Turan Vural E. Vision-Related Quality of Life Before and After Deep Anterior Lamellar Keratoplasty. *Eye Contact Lens* 2018; 44: 144-148. DOI: 10.1097/icl.0000000000000359.

42. In other tables

43. Gothwal VK, Reddy SP, Fathima A, Bharani S, Sumalini R, Bagga DK and Sudharman PM. Assessment of the impact of keratoconus on vision-related quality of life. *Invest Ophthalmol Vis Sci* 2013; 54: 2902-2910. 20130423. DOI: 10.1167/iovs.12-10783.

44. Moschos MM, Gouliopoulos NS, Kalogeropoulos C, Androudi S, Kitsos G, Ladas D, Tsatsos M and Chatziralli I. Psychological Aspects and Depression in Patients with Symptomatic Keratoconus. *J Ophthalmol* 2018; 2018: 7314308. 20180529. DOI: 10.1155/2018/7314308.
